# Bluer in the city: urban male lizards exhibit more intense sexual coloration and lower parasite loads than non‐urban males

**DOI:** 10.1111/1749-4877.12908

**Published:** 2024-09-30

**Authors:** Juan C. GONZÁLEZ‐MORALES, Jimena RIVERA‐REA, Gabriel SUÁREZ‐VARÓN, Elizabeth BASTIAANS, Heliot ZARZA

**Affiliations:** ^1^ Conservation Biology Academic Area, Environmental Science Department, DCBS Metropolitan Autonomous University (UAM) Campus Lerma Lerma de Villada State of Mexico Mexico; ^2^ Amecameca University Center, Autonomous University of the State of Mexico Toluca State of Mexico Mexico; ^3^ Laboratory of Reproductive Morphophysiology, Faculty of Sciences Autonomous University of the State of Mexico Toluca State of Mexico Mexico; ^4^ Biology Department University of New York at Oneonta Oneonta New York USA

**Keywords:** hemoparasites, immune response, *Sceloporus torquatus*, sexual coloration, urbanization

## Abstract

Urbanization is a global phenomenon that involves the transformation of natural areas into urban spaces, thereby subjecting organisms to new selective pressures including a wide variety of pollutants and changes in intra‐ and interspecific interactions. Considering that projections indicate that by the year 2050, 65% of the human population will live in urban areas and that urbanization is a phenomenon with an upward pattern, identifying these phenotypic traits is vital to implementing conservation and management plans for urban fauna. The urban environment may exert different selective pressures on sexually selected traits than more pristine environments, a phenomenon which has been well studied in birds but is less understood in other vertebrates such as lizards, although they are common inhabitants of urban environments. Here, we compare sexual coloration, parasite load, and immune response in *Sceloporus torquatus* lizards in urban and non‐urban environments of Central Mexico. Our study shows that sexual coloration is more saturated (bluer) in male lizards from urban environments, while UV chroma was higher in non‐urban lizards. The average parasite load is lower in urban lizards than in non‐urban lizards, and we found a negative relationship between hemoparasite count and sexual coloration in male lizards from non‐urban environments but not in male lizards from urban environments. Additionally, non‐urban lizards exhibited a higher immune response. In female lizards, sexual coloration differed significantly between urban and non‐urban environments, but parasite load and immune response did not differ. These results may be useful to improve herpetofauna conservation plans in urbanized environments.

## Graphical abstract

The urban environment may exert different selective pressures on sexually selected traits than more pristine environments. Our study shows that sexual coloration is more saturated (bluer) in male lizards from urban environments than in male non‐urban lizards. The average parasite load is lower in urban lizards than in non‐urban lizards. We found a negative relationship between hemoparasite count and sexual coloration in male lizards from non‐urban environments than in urban environments.

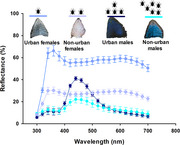

## INTRODUCTION

Urbanization is a worldwide phenomenon that involves converting natural environments to spaces suitable for occupation by high‐density human populations (Marzluff *et al.* 2001; French *et al.* 2014). This process includes infrastructure and building construction, generating the loss of preexisting habitat and reducing biodiversity. Frequently, urban environments are instead dominated by exotic species (Sol *et al.* 2017). In addition, urbanization reduces ecosystem functioning through the presence of impervious surfaces that do not allow water filtration and through a rise in environmental temperatures (Sol *et al.* 2011, 2013; Le Roux *et al.* 2014; Battles *et al.* 2018). Consequently, it gives rise to new challenges for organisms, encompassing chemical, noise, and light pollution, along with habitat loss, fragmentation, and shifts in species interactions (Miranda *et al.* 2013; Sol *et al.* 2013). Currently, 50% of the global human population resides in urban areas, and projections suggest that by 2050, this figure will increase to 65% (United Nations 2019). As urbanization persists as an irreversible trend, it is imperative to understand its impact on the organisms persisting in these environments (McKinney 2008; Dirzo *et al.* 2014).

Urban environments offer habitat for many species of animals. To predict the impact of urbanization on wildlife, various studies have sought to identify crucial traits for successful colonization or persistence in urban environments (French *et al.* 2014; Kang *et al.* 2018; Iglesias‐Carrasco *et al.* 2019; Collins *et al.* 2021). For example, among birds, researchers have underscored the importance of behavioral flexibility and the incorporation of human‐made materials in nest construction (Slabbekoorn & den Boer‐Visser 2006; Suárez‐Rodríguez *et al.* 2013, 2017; Marín‐Gómez & MacGregor‐Fors 2021).

However, knowledge regarding other vertebrates, particularly reptiles, remains somewhat scarce. Studies in this realm have predominantly focused on thermal traits including preferred temperature (Nordberg & Schwarzkopf 2019; Thawley *et al.* 2019), morphological measurements such as body condition or limb length (Putman & Tippie 2020; Gómez‐Benitez *et al.* 2021), immune activity assessed through the heterophile–lymphocyte ratio (French *et al.* 2008; Amdekar *et al.* 2018), dorsal coloration and anti‐predatory behavior (Batabyal & Thaker 2017; Pellitteri‐Rosa *et al.* 2017), and social learning (Kang *et al.* 2018; Batabyal & Thaker 2019).

Sexual selection is a powerful evolutionary force capable of influencing population variability and, consequently, impacting the potential colonization of novel habitats like urban environments (Candolin & Heuschele 2008). The intersexual component of sexual selection frequently relies on the emission of signals, which can manifest visually, chemically, or acoustically (Shuster 2009; Steiger *et al.* 2011; Baeckens *et al.* 2018; Buchinger & Li 2023). In urban settings, sound pollution can limit signals emitted by birds and frogs, disrupting the transmission and reception of vocalizations (Marín‐Gómez & MacGregor‐Fors 2021; Smit *et al.* 2022). These vertebrates have modified the intensity and duration of their signals to avoid overlap with noise pollution, such as increasing volume or altering emission patterns (Slabbekoorn & Peet 2003; Nemeth & Brumm 2010; Francis *et al.* 2011). In contrast, lizards’ sexual signals are predominantly visual, with males exhibiting distinctive colorful features like ventral patches. These features convey vital information, enabling males to compete for resources such as territory, food, or mates (Burtt 1979; Cooper & Burns 1987; Pérez I de Lanuza *et al.* 2013).

An additional effect of urbanization is the modification of species interactions and the host–parasite relationship, which can influence pathogen epidemiology and host susceptibility to infectious diseases (Bradley & Altizer 2007; Delgado & French 2012; Brum *et al.* 2023). Parasites play a significant role in host population dynamics as they draw resources and diminish the fitness of their hosts (Salathé *et al.* 2008; Artim *et al.* 2020; Mendoza‐Roldan *et al.* 2021). They interfere with energy allocation by causing tissue damage and activating the immune system, ultimately affecting host fitness (Meylan *et al.* 2013). In birds, studies have yielded mixed results. Some indicate a lower parasite load in urban areas (Calegaro & Amato 2014; Giraudeau *et al.* 2014), while others demonstrate an increased parasite load (Le Gros *et al.* 2011). In lizards, information is scarce, but prevailing evidence suggests a moderate parasitic increase in urban species (Lazić *et al.* 2017; Thawley *et al.* 2019).

The relationship between parasitic load, sexual coloration, and immune response has been explored in several vertebrates (Hamilton & Zuk 1982; but see Megía‐Palma *et al.* 2021). However, this relationship is rarely studied in urban lizards. Investigating the direction of this relationship in both urban and non‐urban environments may be valuable for comprehending how lizards, and potentially other organisms, modulate the expression of sexual signals, such as sexual coloration, during the colonization of urban habitats. In this study, we examine sexual coloration, immune response, and parasite load in an endemic Mexican lizard that inhabits both urban and non‐urban environments. Our predictions are as follows: (i) lizards from an urban environment will exhibit higher body condition than those from a non‐urban environment; (ii) urban males will display a higher parasite load and less intense blue coloration compared to non‐urban males; and (iii) coloration in urban and non‐urban females will not differ because females do not exhibit the intensely blue sexual coloration typical of males.

*Sceloporus torquatus* is a lizard endemic to central Mexico that in natural environments lives in extrusive igneous rocks in an elevation range between 1600 and 2700 m (González‐Morales *et al.* 2015). In this lizard, males have blue throat and ventral coloration that has been associated with intra‐ and interspecific competition, respectively (Rivera‐Rea *et al.* 2024). The low mobility of this endemic species and the fact that urbanization is encroaching on natural populations make it an appropriate model to examine the effect of urbanization on host–parasite interactions and sexually selected traits.

## MATERIALS AND METHODS

### Ethical statement

This project was conducted under scientific collecting permit number 09/K4‐0002/06/23 from the Mexican Secretaria de Recursos Naturales y Medio Ambiente (SEMARNAT). All field and laboratory procedures were performed in accordance with the ethical guidelines of the Mexican government NOM‐126‐ECOL‐2000.

### Study area, sampling, and animal husbandry

**Figure 1:**
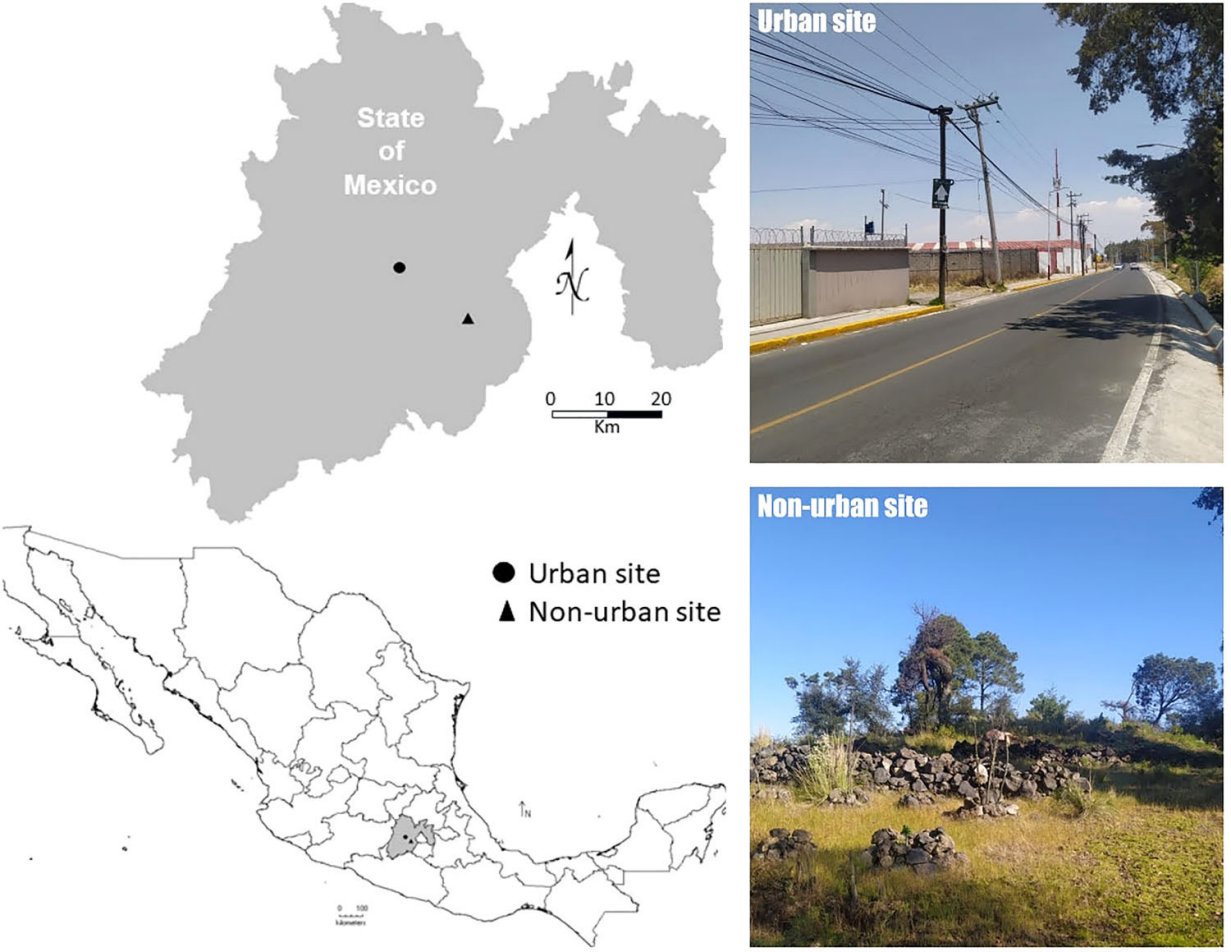
Photographs of a male *Sceloporus torquatus* lizard and the urban and non‐urban study sites.

We studied two populations of *S*. *torquatus* with different levels of urbanization in Central Mexico (Fig. 1). We determined urbanization level according to criteria proposed by Marzluff *et al.* (2001) using residential human and building density as indicators. The urban population was located in Nueva Serratón, Zinacantepec, Mexico State (19°18′42″N, 99°43′41″W) at an elevation of 2642 m. The site consisted of residential housing developments, commercial shops, and busy roads, with lizards primarily using the walls of human houses, human‐constructed rock walls, and rocks as basking sites. The non‐urban population was located in La Joya, San Pedro Techuchulco, Mexico State (19°07′37″N, 99°29′41″W) at an elevation of 2700 m. The site consists of a pine forest without any human buildings nearby, with lizards primarily using extrusive rocks and pine trees as basking sites. The linear distance between the two populations is approximately 33 km. The locations of these sites are sufficiently far enough from each other that gene flow likely does not occur among populations (Sears 2005; Tucker *et al.* 2014).

We sampled adult lizards measuring at least 70 mm snout–vent length (SVL), the minimal adult size for this species (Feria‐Ortiz *et al.* 2001). Since parasite load, ventral coloration, and immune response vary by season in *S*. *torquatus* (Rivera‐Rea *et al.* 2022, 2024), we only captured lizards during the mating season (October–November) by lasso or by hand. We captured 25 urban lizards (15 males and 10 females) and 22 non‐urban lizards (12 males and 10 females). Captured lizards were transported in cotton bags to the Universidad Autónoma Metropolitana Unidad Lerma where they were each kept in an individual terrarium (23 × 41 × 21 cm; width, length, height, respectively) with water and food (*Tenebrio molitor* larvae) *ad libitum* for 7 days. Rocks were placed in one corner and peat moss was used as substrate. During the day (8 AM to 6 PM), heat was provided by a 60 W light bulb (temperature range was 20–35°C in each terrarium). Before starting laboratory procedures, we measured body mass with a balance (precision: ±0.01 g) and SVL with a digital caliper (precision: ±1 mm). We calculated the residual values of a linear regression of log‐mass versus log‐SVL to calculate the body condition index (BCI; Schulte‐Hostedde *et al.* 2005).

### Ectoparasite load

The same researcher (JCGM) conducted an ectoparasite (mite) count using a 20× magnification stereoscope (Zeiss). The examination involved systematically assessing the lizards from head to tail, initially on both the dorsal and ventral surfaces and concluding with the examination of the legs. Body mites (excluding those in pockets) exhibit a correlation with total mites that encompass both pocket and overall body mites (*R* ^2^ = 0.9, *P* < 0.001). In *Sceloporus* lizards, mite pockets are anatomical structures located on each side of the neck that concentrate a large number of mites. These structures have been associated with attracting mites to reduce the damage they cause to other parts of the body (Arnold 1986). Mites of the family Trombiculidae are commonly found on *Sceloporus* species. Distinguished by their red coloration, these mites are easily discernible between the lizard's scales (Guzmán‐Cornejo *et al.* 2018). We categorized the mite count into two groups: mites in pockets and total mites.

### Hemoparasite load

We obtained a blood sample (<10 µL) for the quantification of hemoparasites infecting these lizards, employing methods outlined in Rivera‐Rea *et al.* (2024) for this species. Blood was collected from the coccygeal vein in the tail using sterilized needles, ensuring that incisions avoided the hemipenal bulges. The blood drop obtained was collected with a heparinized capillary (BRAND, micro‐hematocrit tubes, 75 mm). Subsequently, thin‐layer blood smears were prepared, air‐dried, and fixed with methanol for 5 min (Rogier & Landau 1975). These blood smears were stained for 40 min using a 1:10 Giemsa/pH 7.2 buffer solution (Schall 1986). Counting 5000 erythrocytes for each blood smear was performed at 1000× magnification in an area with a homogeneous distribution of red blood cells (Merino & Potti 1995), utilizing a BX41TF microscope (Zeiss). Parasite prevalence of ecto‐ and hemoparasites was calculated as the percentage of infected lizards, while parasite intensity was quantified as the number of parasites per lizard.

### Measuring patch coloration

Because coloration in lizards can change due to body temperature (Langkilde & Boronow 2012; González‐Morales *et al.* 2024), before starting the color quantification, each lizard was kept for 3 h at a temperature of 33°C, which corresponds to the preferred temperature previously recorded for *S. torquatus* in one of the study areas (Rivera‐Rea *et al.* 2018). We quantified the coloration of the lizards’ throat and ventral patches by spectrophotometry within a wavelength range spanning from 300 to 700 nm following a protocol proposed by Rivera‐Rea *et al.* (2024). Measurements were taken from the upper, medial, and lower regions of each blue patch using a Jaz model spectrophotometer equipped with a pulsed xenon light source (all components sourced from Ocean Optics Inc., Dunedin, FL, USA). The light probe was positioned within a black holder to ensure that light readings were 1 mm in diameter, free from ambient light noise, and consistently taken at a distance of 3 mm from the skin surface at a 45° angle. All spectral measurements were referenced against a 99% WS‐1 white reflectance standard.

We processed spectral data in R v.4.2.0 (R Development Core Team 2017) using the “*pavo*” R‐package (Maia *et al.* 2013). We analyzed spectra from the throat and ventral patches separately. We cropped each spectrum between 300 and 700 nm and smoothed it using an interval of 0.2. Then, we extracted colorimetric variables from the spectra of each patch such as luminance, defined as mean brightness; UV chroma, defined as R300–400 nm/R300–700; blue chroma, defined as R400–475 nm/R300–700; and hue, defined as wavelength at the maximum reflectance peak.

### Immune response

We followed the protocol by Rivera‐Rea *et al.* (2022) to assess the immune response in the lizards. We used a phytohemagglutinin (PHA) inoculation test to quantify the local non‐specific inflammatory response of the skin induced by the inoculation of a plant mitogen (Martín *et al.* 2008). PHA influences various T cell‐mediated immune responses and serves as an indicator of heightened immune cell activity. Consequently, PHA is frequently employed in evolutionary ecology studies as a gauge of immune response (Martin *et al.* 2008). For the inoculation, we administered 20‐µL solutions containing 50 mg of PHA in 10 mL of phosphate‐buffered saline (PBS) into the lizards' right hindlimb foot pad. We measured the thickness of the right hindlimb foot pad immediately before and 24 h after PHA inoculation, using the difference as the immune response (IR; Smits *et al.* 1999; Svensson *et al.* 2001). In the left hindlimb foot pad of each lizard, we inoculated an equivalent volume of buffered PBS as a sham control and took identical measurements. The sham control exhibited no significant change in thickness before versus after PBS inoculation (Wilcoxon paired test: *z* = 1.72, *P* = 0.88). All measurements were conducted in triplicate, and the mean was employed in the analyses. The coefficient of variation between measurements was 3.1%. Lizards did not appear to suffer obvious damage during this test, as they remained active and continued normal feeding and thermoregulatory behavior. When we finished collecting data on each lizard, they were kept in captivity for 2 days to allow them to recover before being released at their original collection site.

### Statistical analyses

**Figure 2:**
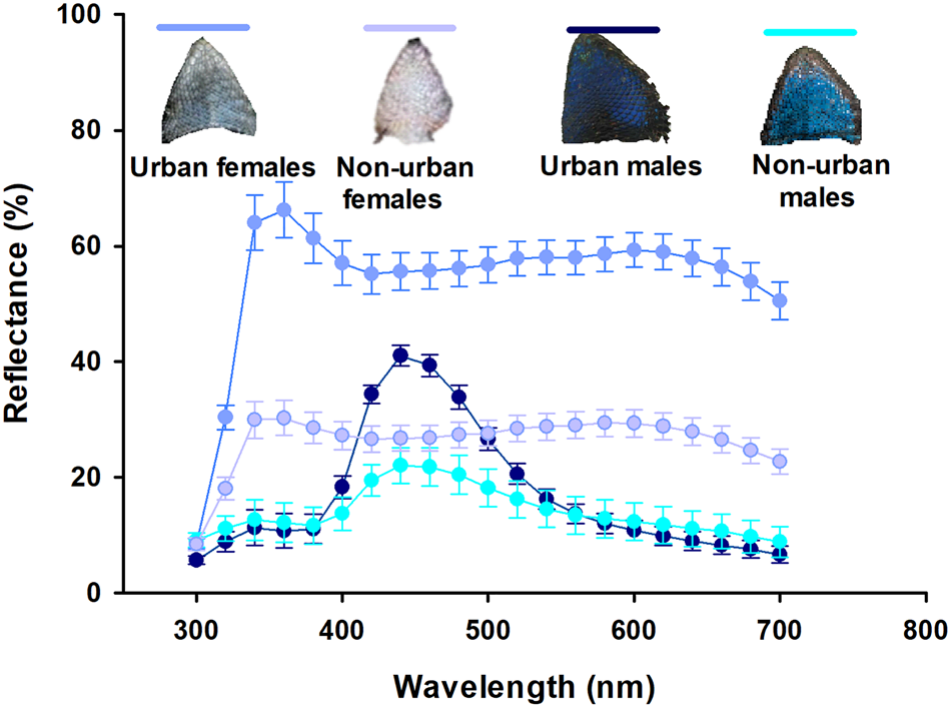
Percentage reflectance by wavelength of throat patches in *Sceloporus torquatus*, separated by habitat and sex.

Before conducting any analyses, we checked the assumptions of normality and homogeneity of variance (Quinn & Keough 2002). We employed the ordinary least square linear model (OLSLM) to compare body mass, SVL, BCI, and immune response using site type (urban or non‐urban) and sex (male or female) as fixed factors. Ectoparasite load was compared using a generalized linear model with a Poisson distribution and log as a linking function. Our data on hemoparasite load included many zero values (22/40 lizards), so we fitted a zero‐inflated model using the “*pscl”* R package (Jackman 2012). We used site and sex as fixed factors and BCI as a continuous covariate. As patch coloration is dimorphic in *S*. *torquatus*, we analyzed spectral measures separately for males and females (Fig. 2). OLSLM was employed to compare luminance, UV, and blue chroma and hue with site type as a fixed factor and BCI as a covariate, but this variable was not significant for any model (results not shown). When we identified significant effects in OLSLM, GLM, or the zero‐inflated model, we conducted Bonferroni post hoc comparisons using the “*emmeans”* R‐package (Lenth 2018). We verified the distributions of all model residuals through visual inspection, utilizing the “*DHARMa”* R‐package (Hartig 2021). Spearman's correlations were employed to assess the relationships between the color components and parasite loads or local immune response. All analyses were performed in R 4.2.0 (R Development Core Team 2017).

## RESULTS

### Body size and body condition

Morphological parameters and immune response of *S. torquatus* in urban and non‐urban habitats are shown in Table 1. Lizards from the urban habitat exhibited larger SVL than non‐urban lizards (*F*_1,43_ = 5.85, *P* = 0.019). Sex and the interaction sex × site type did not have significant effects on SVL (*F*_1,43_ = 2.26, *P* = 0.13 and *F*_1,43_ = 1.67, *P* = 0.20, respectively). The body mass of lizards from urban habitats was heavier than lizards from non‐urban habitats (*F*_1,43_ = 4.85, *P* = 0.033). Sex and the interaction site type × sex did not significantly affect body mass (*F*_1,43_ = 2.98, *P* = 0.09 and *F*_1,43_ = 0.45, *P* = 0.50, respectively). No significant differences were observed in BCI across site type (*F*_1,43_ = 0.008, *P* = 0.99), sex (*F*_1,43_ = 0.007, *P* = 0.99), or their interaction (*F*_1,43_ = 0.007, *P* = 0.99).

**Table 1:** Morphological parameters and immune response of *Sceloporus torquatus* from urban versus non‐urban lizards

	Urban		Non‐urban	
	Males	Females	Males	Females
Body mass (g)	31.2 ± 2.4	29.1 ± 1.71	27.7 ± 2.32	22.6 ± 1.43
Snout vent–length (mm)	91.7 ± 2.91	92.4 ± 1.67	88.6 ± 2.36	81.9 ± 1.44
Body condition index (BCI)	1.33 × 10^−6^ ± 0.01	10.00 × 10^−6^ ± 0.01	−2.89 × 10^−19^ ± 0.01	1.60× 10^−5^ ± 0.01
Immune response (mm)	0.0125 ± 0.002	0.0217 ± 0.001	0.0267 ± 0.002	0.0268 ± 0.003

Mean ± standard error of the mean (SEM).

### Parasite load and immune response

Mite infestation in pockets differed by site type (χ^2^ = 1019.45, df = 1, *P* < 0.001; Table 2), sex (χ^2^ = 22.86, df = 1, *P* < 0.001), and the interaction site type × sex (χ^2^ = 68.01, df = 1, *P* < 0.001). Urban males exhibited higher mite counts in pockets than urban females (*z* test = −4.73, *P* < 0.001) but lower than non‐urban males (*z* test = 20.39, *P* < 0.001), while, non‐urban males had higher mite counts in pockets than non‐urban females (*z* test = 4.72, *P* < 0.001). Total mite counts also differed by site type (χ^2^ = 290.11, df = 1, *P* < 0.001), sex (χ^2^ = 548.11, *gl* = 1, *P* < 0.001), and the interaction site type × sex (χ^2^ = 20.11, df = 1, *P* < 0.001). Urban males had higher total mites than urban females (*z* test = −11.37, *P* < 0.001) but lower than non‐urban males (*z* test = 32.73, *P* < 0.001), while males from non‐urban habitats had higher total mite counts than non‐urban females (*z* test = −22.74, df = 1, *P* < 0.001). Hemoparasite load did not differ by site type (χ^2^ = 0.08, df = 1, *P* = 0.87) but did differ by sex (χ^2^ = 17.75, *gl* = 1, *P* < 0.001). Males had higher hemoparasite load than females (*z* test = 2.79, *P* = 0.005). The interaction site type × sex was not significant (χ^2^ = 3.57, df = 1, *P* = 0.07). Urban male lizards with higher hemoparasite loads had lower body mass than males with fewer hemoparasites (*R* ^2^ = −0.58, *P* = 0.02), but in non‐urban lizards, we did not find this relationship (*R* ^2^ = 0.009, *P* = 0.98). Immune response varied with site type; urban lizards exhibited lower immune responses than non‐urban lizards (*F*_1,42_ = 19.38, *P* < 0.001). Immune response varied by sex; males had lower immune responses than females (*F*_1,42_ = 6.50, *P* = 0.015). The interaction site type × sex was not significant (*F*_1,42_ = 2.02, *P* = 0.16). In female non‐urban lizards, the immune response was significantly negatively related to hemoparasite load (*R* ^2^ = −0.9, *P* < 0.001); lizards with higher hemoparasite loads had lower immune response. This relationship was not found in male non‐urban lizards (*R* ^2^ = 0.28, *P* = 0.37) or in urban lizards (*R* ^2^ = −0.18, *P* = 0.53)

**Table 2:** Parasite loads of *Sceloporus torquatus* from urban and non‐urban habits by sex

		Mean ± SEM	Range	Prevalence
Mites pockets				
Urban	Male	4.87 ± 2.2	0–30	53.3%
	Female	0.3 ± 0.2	0–2	20%
Non‐urban	Male	61.7 ± 22.4	1–215	100%
	Female	81.3 ± 20.3	8–160	100%
Total mites				
Urban	Male	183 ± 42	5–536	100%
	Female	124 ± 28.2	33–280	100%
Non‐urban	Male	400 ± 80.8	40–1046	100%
	Female	224± 37	43–400	100%
Hemoparasites				
Urban	Male	1.73 ± 0.7	0–9	46.6%
	Female	0.9 ± 0.6	0–6	30%
Non‐urban	Male	5.58 ± 2.39	0–23	66.6%
	Female	1.9 ± 0.5	0–5	70%

Intensity values are summarized as mean ± standard error of the mean (SEM) and range (minimum–maximum). Prevalence represents the percentage of lizards in each category with each kind of parasite infestation.

### Male coloration

**Figure 3:**
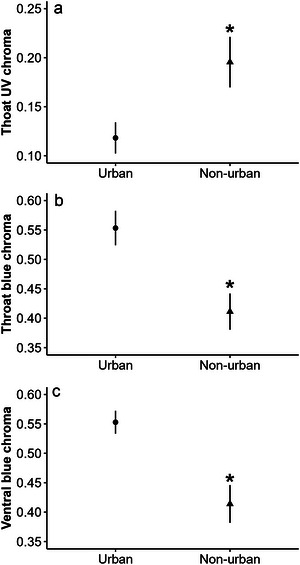
Chroma ± standard error of the mean (SEM) of *Sceloporus torquatus* males from urban versus non‐urban habitats: (a) throat UV chroma, (b) throat blue chroma, and (c) ventral blue chroma. Asterisks show differences by site.

**Figure 4:**
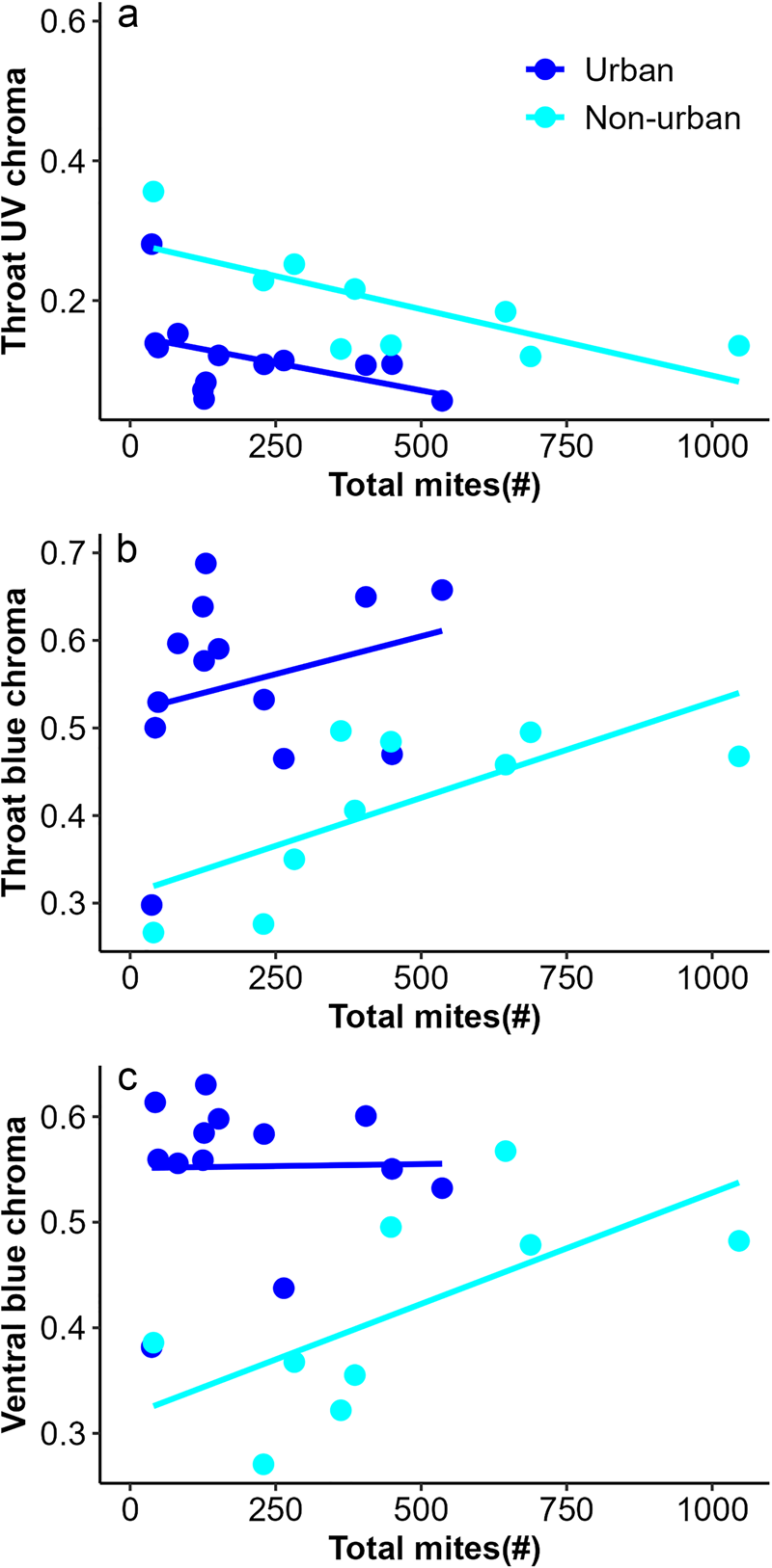
Relationship between chroma and total mites in *Sceloporus torquatus* from urban and non‐urban sites: (a) throat UV chroma, (b) throat blue chroma, and (c) ventral blue chroma.

Throat luminance did not differ between urban and non‐urban males (*F*_1,23_ = 2.90, *P* = 0.10). Urban lizards exhibited lower values of throat UV chroma than non‐urban lizards (*F*_1,23_ = 5.26, *P* = 0.03; Fig. 3a) but higher values of throat blue chroma than non‐urban lizards (*F*_1,23_ = 12.78, *P* = 0.001; Fig. 3b). Throat hue did not differ between urban and non‐urban lizards (*F*_1,23_ = 0.11, *P* = 0.73). In non‐urban lizards, we observed a marginally significant and negative relationship between throat UV chroma and mites in pockets (*R* ^2^ = −0.68, *P* = 0.05); lizards with more mites had lower UV chroma. This relationship was not observed in urban lizards (*R* ^2^ = −0.25, *P* = 0.4). For urban lizards, total mites were significantly and negatively related to UV chroma (*R* ^2^ = −0.66, *P* = 0.01; Fig. 4a); lizards with higher total mites had lower UV chroma values. This relationship was not present in non‐urban lizards (*R* ^2^ = −0.48, *P* = 0.12). In non‐urban lizards, total mites were significantly and positively related to blue chroma (*R* ^2^ = 0.8, *P* = 0.003; Fig. 4b); lizards with higher total mites had higher blue chroma. In urban lizards, this relationship was not present (*R* ^2^ = 0.37, *P* = 0.2).

Ventral luminance and ventral UV chroma in urban lizards did not differ from non‐urban lizards (*F*_1,23_ = 0.95, *P* = 0.33 and *F*_1,23_ = 3.02, *P* = 0.09, respectively). Urban lizards had a higher blue chroma than non‐urban lizards (*F*_1,23_ = 12.69, *P* = 0.001; Fig. 3c). Ventral hue did not differ between urban and non‐urban lizards (*F*_1,23_ = 0.006, *P* = 0.93). In non‐urban lizards, we found a significant and negative relationship between total mites and ventral UV chroma (*R* ^2^ = −0.62, *P* = 0.03); lizards with higher total mites had lower UV chroma. This relationship was not observed in urban lizards (*R* ^2^ = −0.17, *P* = 0.55). Similarly, a significant and negative relationship was found between hemoparasite load and ventral UV chroma in non‐urban lizards (*R* ^2^ = −0.61, *P* = 0.03); lizards with higher hemoparasite loads had lower UV chroma. This relationship was not observed in urban lizards (*R* ^2^ = 0.07, *P* = 0.8). Moreover, a significant and negative relationship was identified between total mites and ventral blue chroma in non‐urban lizards (*R* ^2^ = −0.62, *P* = 0.03; Fig. 3c); lizards with higher total mites had lower blue chroma. This relationship was not observed in urban lizards (*R* ^2^ = 0.02, *P* = 0.95).

### Female coloration

Female urban lizards exhibited higher throat luminance values compared to non‐urban lizards (*F*_1,17_ = 55.07, *P* < 0.001). No differences were found in UV chroma (*F*_1,17_ = 0.009, *P* = 0.92) or blue chroma (*F*_1,17_ = 3.03, *P* = 0.09) between urban and non‐urban lizards. However, urban lizards had a lower throat hue than their non‐urban lizards (*F*_1,17_ = 6.69, *P* = 0.019). A marginally significant relationship was observed between body mites and throat UV chroma in urban lizards (*R* ^2^ = −0.64, *P* = 0.05); lizards with higher total mites had lower UV chroma values. Interestingly, this relationship was not found in non‐urban lizards (*R* ^2^ = −0.38, *P* = 0.36).

Urban lizards exhibited higher ventral luminance than non‐urban lizards (*F*_1,17_ = 38.01, *P* < 0.001). However, no differences were observed in ventral UV and blue chroma based on habitat (*F*_1,17_ = 0.005, *P* = 0.93 and *F*_1,17_ = 0.06, *P* = 0.79, respectively). Ventral hue also did not show differences based on habitat (*F*_1,17_ = 0.52, *P* = 0.48). Ventral spectral variables were not related to any parasite load measure (see Table S1, Supporting Information).

## DISCUSSION

This is the first study to examine the effect of urbanization on sexual color expression in lizards, aiming to analyze the interplay between sexual coloration, parasite load, and immune response in both urban and non‐urban lizard populations. Our results reveal that urban males of *S*. *torquatus* had bluer ventral chroma, lower parasite load, and lower immune response compared to their counterparts in natural habitats. Furthermore, male lizards in the natural environment demonstrated a negative correlation between blue chroma and parasite load, a relationship not observed in urban lizards. However, urban male lizards exhibited a negative correlation between UV chroma and total mites, a relationship not observed in non‐urban lizards. Moreover, urban females display elevated ventral luminance compared to non‐urban females, although this characteristic was not correlated with either parasite load or immune response.

In this study, urbanization was linked to an increase in body size, but body condition did not vary between urban and non‐urban lizards. The increase in body size in urban lizards has been reported in other species and is commonly linked to factors such as habitat composition, food availability, and divergent predator communities (Prosser *et al.* 2006; Lazić *et al.* 2017; Gómez‐Benítez *et al.* 2021). Habitat composition significantly influences lizard behavior and performance. In open habitats, lizards may counter higher predator exposure with enhanced running speed, often tied to increased limb length (Garland & Losos 1994; Cooper 1997; Vanhooydonck & Van Damme 1999). However, this relationship is not a common pattern across all lizard species (Prosser *et al.* 2006; González‐Morales *et al.* 2021). Certain species, like *Sceloporus occidentalis* and *Aspidoscelis costatus* exhibit morphological adaptations based on the anthropogenic structures they encounter (Putman *et al.* 2019; Gómez‐Benítez *et al.* 2021). Conversely, urban habitats are often characterized by reduced predator diversity, potentially leading to an increase in lizard body size due to the relaxation of selective pressures (Eötvös *et al.* 2018). The body condition of lizards closely correlates with health and fitness, serving as an indicator of nutritional and physical well‐being (Labocha *et al.* 2014; Lazić *et al.* 2017). Contrary to our initial prediction, urban lizards did not exhibit a higher body condition index than their non‐urban counterparts. Putman and Tippie (2020) found that urban lizards showed a higher body condition index compared to those in natural or non‐urban habitats. Several non‐exclusive hypotheses have been proposed to explain this discrepancy, ranging from increased foraging time to reduced predation pressure (Iglesias *et al.* 2012; Eötvös *et al.* 2018; Putman & Tippie 2020). In our study, we did not observe differences in the body condition index. This outcome could be attributed to the fact that we captured lizards during the fall in Mexico, a season when this species is in the reproductive phase. The associated costs linked to courtship behavior and territory defense during this period could limit foraging time (Rivera‐Rea *et al.* 2022, 2024). To understand these lizards’ physiology better, we recommend studies quantifying the quantity and quality of prey in urban and non‐urban environments. This information would shed light on how these factors may influence lizards' fat reserves.

Our second prediction was not fulfilled, because urban lizards had fewer ecto‐ and hemoparasites than non‐urban lizards. The parasite–host relationship is not immune to the impacts of urbanization, and outcomes can vary across different taxa, resulting in either an increase or decrease in parasite intensity (Martin *et al.* 2010). In our study, we observed that urban lizards harbor fewer ecto‐ and hemoparasites compared to their non‐urban counterparts. Factors such as habitat fragmentation, reduced environmental humidity, and increased environmental temperatures in urban settings may contribute to a decreased probability of parasite survival (Schall 1992; Dobson *et al.* 1992; Riley *et al.* 2014; Thawley *et al.* 2019). Additionally, the absence of intermediate hosts in the life cycle of parasites could further diminish the likelihood of infecting urban lizards (Civitello *et al.* 2015). In natural or preserved environments, dense vegetation cover and high relative humidity elevate the probability of infection in lizards, conditions rarely found in urban areas (Rivera‐Rea *et al.* 2022). While the specific mechanisms by which urban lizards manage to evade high parasite loads remain unclear, there is mounting evidence of parasites' detrimental effects on the quality of signals in vertebrates. Generally, parasites activate the immune system, triggering a resource reallocation that may influence other traits, such as the expression of sexual signals (López *et al.* 2009; Plasman *et al.* 2015; Rivera‐Rea *et al.* 2024). Our results indicate that lizards from natural habitats exhibit lower expression of the blue coloration in the ventral zone, coupled with a higher parasite load, possibly indicative of maintaining a robust immune response. Interestingly, lizards from natural areas displayed a higher number of parasites in the mite pockets compared to urban lizards. These mite pockets, characterized by skin invagination, have been associated with effective mite infestation control (Arnold 1986; Salvador *et al.* 1999). The skin within these structures is hyperplastic and resilient, featuring an accumulation of lymphoid cells that facilitate rapid tissue repair and infection relief (Arnold 1986). Future studies should explore whether these structures influence the honesty of visual signals in lizards.

Vertebrates, including lizards, employ two distinct pigment types to generate sexual coloration, and these may be differentially influenced by urbanization (McGraw 2006a, 2006b). Coloration based on carotenoids cannot be produced endogenously by vertebrates and must be acquired through diet, making it a reliable indicator of body condition and foraging efficiency (McGraw 2006a; Biard *et al.* 2017). In contrast, structural (or melanin) coloration can be synthesized endogenously by animals and is considered less condition‐dependent and more genetically controlled than carotenoid coloration (McGraw 2006b; Roulin 2016). However, structural coloration is energetically costly to produce, limiting its expression to individuals in good condition (McGraw 2006b; Roulin 2016). The throat and ventral patch coloration of *S*. *torquatus* exemplifies structural coloration and has been linked to high male quality (Rivera‐Rea *et al.* 2024). In this study, male lizards from urban sites exhibited a higher blue chroma (i.e. more intense blue coloration) than non‐urban males. Urban pollution, particularly with metals, can favor darker or bluer coloration phenotypes. Metal pollution may drive blue coloration by increasing circulating testosterone, and some metals such as zinc and calcium can also serve as micronutrients supporting melanogenesis (Laskey & Phelps 1991; Dauwe & Eens 2008; Pacyna *et al.* 2018). This study is the first to test the interaction of sexual coloration and immune functions in urban and rural lizards for the first time. However, it has been a more recurrent subject in other vertebrates, such as birds (Grunst *et al.* 2020). In great tits (*Parus major*), anthropogenic pollution adversely affects carotenoid coloration but not structural coloration (Grunst *et al.* 2020). Generally, structural coloration is more prevalent in urban sites, as reported by Leveau (2021).

An additional explanation for our results is that urban lizards, with lower parasite loads, may allocate more resources to coloration, resulting in more intense (blue) coloration (Rivera‐Rea *et al.* 2024). In contrast, non‐urban lizards, with a higher number of parasites and a robust immune response, may compromise the expression of the blue color (López *et al.* 2009; Megía‐Palma *et al.* 2021). Our results indicate differential correlations between ectoparasite load and color components in urban versus non‐urban environments. In urban lizards, ectoparasite load is related to UV chroma, while in non‐urban lizards, it is related to blue chroma. Selective pressures differ between urban and non‐urban environments (Sol *et al.* 2011, 2013; Le Roux *et al.* 2014; Battles *et al.* 2018); factors such as predation, parasite type, and species, and pollutants can greatly influence the development of sexual coloration (McLean *et al.* 2015; Megía‐Palma *et al.* 2016, 2018; Marín‐Gómez & MacGregor‐Fors 2021; Smit *et al.* 2022). Further research into signal expression in lizards from urban and non‐urban environments is needed to understand better the causes and consequences of the shifts in color components.

An alternative explanation for the observed phenotypic differences between urban and non‐urban lizards could be intrinsic variation between populations unrelated to urban environmental pressures (Smyth *et al.* 2014), although we attempted to minimize this possibility by choosing locations that were geographically close together and located at similar elevations. Another possibility is that only those lizards with high levels of resistance to parasites succeed in colonizing urban environments, founding populations with a high level of local adaptation to parasites, which may subsequently affect the sexual signals present in those populations (Fricke *et al.* 2010; Gómez‐Llano *et al.* 2020). Finally, the structural arrangement producing the blue color could be subject to developmental modifications, independent of urbanization effects (Jeon *et al.* 2021; Radovanović *et al.* 2023). Future studies should include replicate populations to determine if this variation is consistent between environments (Macotela *et al.* 2023) and utilize common garden experiments to rear lizards from birth under controlled conditions to verify whether color expression remains constant

Our third prediction rested on the expectation that females would not show differences in ventral coloration. This expectation arises from the species‐specific feature where males are the primary developers of ventral blue coloration. Our result showed that female urban lizards exhibited higher throat and ventral patch luminance than non‐urban female lizards. Research related to sexual coloration often focuses on males, who are generally the ones who express or exaggerate these traits. However, females in many species where males are more brightly colored also use coloration as an indicator of quality and for communication (Kraaijeveld *et al.* 2007). Females with less intense orange coloration in Alpine newt (*Ichthyosaura alpestris*) lay eggs more slowly compared to females with more intense orange coloration (Lüdtke & Foerster 2019), while in other species such as convict cichlids (*Cichlasoma nigrofasciatum*), coloration has been related to levels of aggressiveness (Beeching *et al.* 1998). In our study, none of the color components was related to the traits we evaluated; however, this does not mean that it does not have a function as such. In juvenile lizards of *Sceloporus undulatus*, females exhibit a coloration similar to that of males, albeit less intense. This phenomenon has been associated with low levels of corticosterone, which may have implications for the quality of offspring (Assis *et al.* 2021), so it is necessary to reconsider what this female coloration function could be in future studies.

In conclusion, our results indicate significant differences in color expression, parasite load, and immune response between urban and non‐urban lizards. Examining a broader range of species and phenotypic traits in reptiles is essential to understanding the patterns facilitating their colonization and persistence in urban environments. This knowledge will enhance both conservation strategies and urban development plans, ensuring better integration of wildlife considerations in city planning.

## CONFLICT OF INTEREST STATEMENT

The authors declare no competing interests.

## Supporting information




**Table S1** Spearman correlation results between female ventral spectral variables and parasite load

## References

[inz212908-bib-0001] Amdekar MS , Kakkar A , Thaker M (2018). Measures of health provide insights into the coping strategies of urban lizards. Frontiers in Ecology and Evolution 6, 128.

[inz212908-bib-0002] Arnold EN (1986). Mite pockets of lizards, a possible means of reducing damage by ectoparasites. Biological Journal of the Linnean Society 29, 1–21.

[inz212908-bib-0003] Artim JM , Nicholson MD , Hendrick GC , Brandt M , Smith TB , Sikkel PC (2020). Abundance of a cryptic generalist parasite reflects degradation of an ecosystem. Ecosphere 11, e03268

[inz212908-bib-0004] Assis BA , Avery JD , Tylan C , Engler HI , Earley RL , Langkilde T (2021). Honest signals and sexual conflict: Female lizards carry undesirable indicators of quality. Ecology and Evolution 11, 7647–7659. 34188841 10.1002/ece3.7598 PMC8216924

[inz212908-bib-0005] Baeckens S , Martín J , García‐Roa R , Van Damme R (2018). Sexual selection and the chemical signal design of lacertid lizards. Zoological Journal of the Linnean Society 183, 445–457.

[inz212908-bib-0006] Batabyal A , Thaker M (2017). Signalling with physiological colours: High contrast for courtship but speed for competition. Animal Behaviour 129, 229–236.

[inz212908-bib-0007] Batabyal A , Thaker M (2019). Social coping styles of lizards are reactive and not proactive in urban areas. General and Comparative Endocrinology 270, 67–74. 30336119 10.1016/j.ygcen.2018.10.007

[inz212908-bib-0008] Battles AC , Moniz M , Kolbe JJ (2018). Living in the big city: Preference for broad substrates results in niche expansion for urban *Anolis* lizards. Urban Ecosystems 21, 1087–1095.

[inz212908-bib-0009] Beeching SC , Gross SH , Bretz HS , Hariatis E (1998). Sexual dichromatism in convict cichlids: The ethological significance of female ventral coloration. Animal Behavior 56, 1021–1026. 10.1006/anbe.1998.0868 9790714

[inz212908-bib-0010] Biard C , Brischoux F , Meillère A *et al.* (2017). Growing in cities: An urban penalty for wild birds? A study of phenotypic differences between urban and rural great tit chicks (*Parus major*). Frontiers in Ecology and Evolution 5, 79.

[inz212908-bib-0011] Bradley CA , Altizer S (2007). Urbanization and the ecology of wildlife diseases. Trends in Ecology and Evolution 22, 95–102. 17113678 10.1016/j.tree.2006.11.001 PMC7114918

[inz212908-bib-0012] Brum PHR , Gonçalves SRA , Strüssmann C , Teixido AL (2023). A global assessment of research on urban ecology of reptiles: Patterns, gaps and future directions. Animal Conservation 26, 1–13.

[inz212908-bib-0013] Buchinger TJ , Li W (2023). Chemical communication and its role in sexual selection across Animalia. Communications Biology 6, 1178. 37985853 10.1038/s42003-023-05572-w PMC10662023

[inz212908-bib-0014] Burtt EH (1979). The Behavioral Significance of Color. Garland Press, New York

[inz212908-bib-0015] Calegaro‐Marques C , Amato SB (2014). Urbanization breaks up host‐parasite interactions: A case study on parasite community ecology of rufous‐bellied thrushes (*Turdus rufiventris*) along a rural‐urban gradient. PLoS ONE 9, e103144. 25068271 10.1371/journal.pone.0103144 PMC4113371

[inz212908-bib-0016] Candolin U , Heuschele J (2008). Is sexual selection beneficial during adaptation to environmental change? Trends in Ecology and Evolution 23, 446–452. 18582989 10.1016/j.tree.2008.04.008

[inz212908-bib-0017] Civitello DJ , Cohen J , Fatima H *et al*. (2015). Biodiversity inhibits parasites: Broad evidence for the dilution effect. PNAS 112, 8667–8671. 26069208 10.1073/pnas.1506279112 PMC4507196

[inz212908-bib-0018] Collins MK , Magle SB , Gallo T (2021). Global trends in urban wildlife ecology and conservation. Biological Conservation 261, 109236.

[inz212908-bib-0019] Cooper WE (1997). Escape by a refuging prey, the Broad‐Headed Skink (*Eumeces laticeps*). Canadian Journal of Zoology 75, 943–947.

[inz212908-bib-0020] Cooper WE , Burns N (1987). Social significance of ventrolateral coloration in the fence lizard, *Sceloporus undulatus* . Animal Behavior 35, 526–532.

[inz212908-bib-0021] Dauwe T , Eens M (2008). Melanin‐and carotenoid‐dependent signals of great tits (*Parus major*) relate differently to metal pollution. Naturwissenschaften 95, 969–973. 18506415 10.1007/s00114-008-0400-1

[inz212908-bib-0022] Delgado VCA , French K (2012). Parasite–bird interactions in urban areas: Current evidence and emerging questions. Landscape and Urban Planning 105, 5–14.

[inz212908-bib-0023] Dirzo R , Young HS , Galetti M , Ceballos G , Isaac NJB , Collen B (2014). Defaunation in the anthropocene. Science 345, 401–406. 25061202 10.1126/science.1251817

[inz212908-bib-0024] Dobson AP , Pacala SV , Roughgarden JD , Carper ER , Harris EA (1992). The parasites of *Anolis* lizards in the northern Lesser Antilles: I. Patterns of distribution and abundance. Oecologia 91, 110–117. 28313381 10.1007/BF00317248

[inz212908-bib-0025] Eötvös CB , Magura T , Lövei GL (2018). A meta‐analysis indicates reduced predation pressure with increasing urbanization. Landscape and Urban Planning 180, 54–59.

[inz212908-bib-0026] Feria‐Ortiz M , Nieto‐Montes de Oca A , Salgado‐Ugarte IH (2001). Diet and reproductive biology of the viviparous lizard *Sceloporus torquatus torquatus* (Squamata: Phrynosomatidae). Journal of Herpetology 35, 104–112.

[inz212908-bib-0027] Francis CD , Ortega CP , Cruz A (2011). Noise pollution filters bird communities based on vocal frequency. PLoS ONE 6, e27052. 22096517 10.1371/journal.pone.0027052 PMC3212537

[inz212908-bib-0028] French SS , Fokidis HB , Moore MC (2008). Variation in stress and innate immunity in the tree lizard (*Urosaurus ornatus*) across an urban–rural gradient. Journal of Comparative Physiology B 178, 997–1005. 10.1007/s00360-008-0290-8 PMC2774757 18594834

[inz212908-bib-0029] French SS , Webb AC , Hudson SB , Virgin EE (2014). Town and country reptiles: A review of reptilian responses to urbanization. Integrative and Comparative Biology 58, 948–966. 10.1093/icb/icy052 29873730

[inz212908-bib-0030] Fricke JM , Vardo‐Zalik AM , Schall JJ (2010). Geographic genetic differentiation of a malaria parasite, *Plasmodium mexicanum*, and its lizard host, *Sceloporus occidentalis* . Journal of Parasitology 96, 308–313. 19916631 10.1645/GE-2304.1

[inz212908-bib-0031] Garland T , Losos JB (1994). Ecological morphology of locomotor performance in squamate reptiles. In: Wainwright PC , Reilly SM, eds. Ecological Morphology: Integrative Organismal Biology. University of Chicago Press, Chicago, IL, pp. 240–302.

[inz212908-bib-0032] Giraudeau M , Mousel M , Earl S , McGraw K (2014). Parasites in the city: Degree of urbanization predicts poxvirus and coccidian infections in house finches (*Haemorhous mexicanus*). PLoS ONE 9, e86747. 24503816 10.1371/journal.pone.0086747 PMC3913573

[inz212908-bib-0033] Gómez‐Benitez A , Walker JM , López‐Moreno AE , Hernández‐Gallegos O (2021). The influence of urbanization on morphological traits in the Balsas Basin Whiptail lizard (*Aspidoscelis costatus costatus*). Urban Ecosystems 24, 327–333.

[inz212908-bib-0034] Gómez‐Llano M , Narasimhan A , Svensson EI (2020). Male‐male competition causes parasite‐mediated sexual selection for local adaptation. The American Naturalist 196, 344–354. 10.1086/710039 32814001

[inz212908-bib-0035] González‐Morales JC , Quintana E , Díaz‐Albiter H , Guevara‐Fiore P , Fajardo V (2015). Is erythrocyte size a strategy to avoid hypoxia in Wiegmann's Torquate Lizards (*Sceloporus torquatus*)? Field evidence. Canadian Journal of Zoology 93, 377–382.

[inz212908-bib-0036] González‐Morales JC , Rivera‐Rea J , Moreno‐Rueda G , Bastiaans E , Castro‐López M , Fajardo V (2021). Fast and dark: The case of Mezquite lizards at extreme altitude. Journal of Thermal Biology 102, 103115. 34863479 10.1016/j.jtherbio.2021.103115

[inz212908-bib-0037] González‐Morales JC , Rivera‐Rea J , Moreno‐Rueda G , Plasman M , Quintana E , Bastiaans E (2024). Seasonal and altitudinal variation in dorsal skin reflectance and thermic rates in a high‐altitude montane lizard. International Journal of Biometeorology 68, 1421–1435. 38652160 10.1007/s00484-024-02677-7

[inz212908-bib-0038] Grunst ML , Grunst AS , Pinxten R , Bervoets L , Eens M (2020). Carotenoid‐but not melanin‐based plumage coloration is negatively related to metal exposure and proximity to the road in an urban songbird. Environmental Pollution 256, 113473. 31679871 10.1016/j.envpol.2019.113473

[inz212908-bib-0039] Guzmán‐Cornejo C , García‐Prieto L , Zúñiga‐Vega J (2018). First quantitative data on the ectoparasites mites of *Sceloporus torquatus* from the Ecological Reserve of Pedregal de San Angel in Central Mexico. Acarologia 55, 868–874.

[inz212908-bib-0040] Hamilton WD , Zuk M (1982). Heritable true fitness and bright birds: A role for parasites? Science 218, 384–387. 7123238 10.1126/science.7123238

[inz212908-bib-0041] Hartig F (2021). DHARMa: residual diagnostics for hierarchical (multi‐level/mixed) regression models, R Package Version 0.4.4. Available from URL: https://cran.r‐project.org/web/packages/DHARMa/index.html

[inz212908-bib-0042] Iglesias S , Tracy C , Bedford G , Christian K (2012). Habitat differences in body size and shape of the Australian agamid lizard, *Lophognathus temporalis* . Journal of Herpetology 46, 297–303.

[inz212908-bib-0043] Iglesias‐Carrasco M , Duchêne DA , Head ML , Møller AP , Cain K (2019). Sex in the city: Sexual selection and urban colonization in passerines. Biology Letters 15, 20190257. 31480935 10.1098/rsbl.2019.0257 PMC6769144

[inz212908-bib-0044] Jackman S (2012). pscl: classes and methods for R developed in the political science computational laboratory, R Package Version 1.04.1. Stanford University, Stanford, CA. Available from URL: http://pscl.stanford.edu/.

[inz212908-bib-0045] Jeon DJ , Paik S , Ji S , Yeo JS (2021). Melanin‐based structural coloration of birds and its biomimetic applications. Applied Microscopy 51, 14. 34633588 10.1186/s42649-021-00063-w PMC8505553

[inz212908-bib-0046] Kang F , Goulet CT , Chapple DC (2018). The impact of urbanization on learning ability in an invasive lizard. Biological Journal of the Linnean Society 123, 55–62.

[inz212908-bib-0047] Kraaijeveld K , Kraaijeveld‐Smit FJL , Komdeur J (2007). The evolution of mutual ornamentation. Animal Behavior 74, 657–677.

[inz212908-bib-0048] Labocha MK , Schutz H , Hayes JP (2014). Which body condition index is best? Oikos 123, 111–9.

[inz212908-bib-0049] Langkilde T , Boronow KE (2012). Hot boys are blue: Temperature‐dependent color change in male eastern fence lizards. Journal of Herpetology 46, 461–465.

[inz212908-bib-0050] Laskey JW , Phelps PV (1991). Effect of cadmium and other metal cations on in vitro Leydig cell testosterone production. Toxicology and applied pharmacology 108, 296–306. 1850171 10.1016/0041-008x(91)90119-y

[inz212908-bib-0051] Lazić MM , Carretero MA , Živković U , Crnobrnja‐Isailović J (2017). City life has fitness costs: Reduced body condition and increased parasite load in urban common wall lizards, *Podarcis muralis* . Salamandra 53, 10–17.

[inz212908-bib-0052] Le Gros A , Stracey CM , Robinson SK (2011). Associations between Northern Mockingbirds and the parasite *Philornis porteri* in relation to urbanization. The Wilson Journal of Ornithology 123, 788–796.

[inz212908-bib-0053] Le Roux DS , Ikin K , Lindenmayer DB , Blanchard W , Manning AD , Gibbons P (2014). Reduced availability of habitat structures in urban landscapes: implications for policy and practice. Landscape and Urban Planning 125, 57–64.

[inz212908-bib-0054] Lenth R (2018). emmeans: estimated marginal means, aka least‐squares means. v 1.2.2. Available from URL: https://rvlenth.github.io/emmeans/

[inz212908-bib-0055] Leveau L (2021). United colours of the city: A review about urbanisation impact on animal colours. Austral Ecology 46, 670–679.

[inz212908-bib-0056] López P , Gabirot M , Martín J (2009). Immune challenge affects sexual coloration of male Iberian wall lizards. Journal of Experimental Zoology A 311, 96–104 10.1002/jez.505 18942109

[inz212908-bib-0057] Lüdtke DU , Foerster K (2019). A female color ornament honestly signals fecundity. Frontiers in Ecology and Evolution 7, 432.

[inz212908-bib-0058] Macotela L , Naya DE , González‐Morales JC , Anaya M , Fajardo V , Manjarrez J (2023). Altitudinal variation in organ mass from three mountain systems: The case of mesquite lizard *Sceloporus grammicus* . Comparative Biochemistry and Physiology Part A 281, 111426. 10.1016/j.cbpa.2023.111426 37059292

[inz212908-bib-0059] Maia R , Eliason CM , Bitton PP , Doucet SM , Shawkey MD (2013). pavo: An R package for the analysis, visualization and organization of spectral data. Methods in Ecology and Evolution 4, 906–913.

[inz212908-bib-0060] Marín‐Gómez OH , MacGregor‐Fors I (2021). A global synthesis of the impacts of urbanization on bird dawn choruses. Ibis 163, 1133–1154.

[inz212908-bib-0061] Martín J , Amo L , López P (2008). Parasites and health affect multiple sexual signals in male common wall lizards, *Podarcis muralis* . The Science of Nature 95, 293–300. 10.1007/s00114-007-0328-x 18060654

[inz212908-bib-0062] Martin LB , Hopkins WA , Mydlarz LD , Rohr JR (2010). The effects of anthropogenic global changes on immune functions and disease resistance. Annals of the New York Academy Sciences 1195, 129–48. 10.1111/j.1749-6632.2010.05454.x 20536821

[inz212908-bib-0063] Marzluff JM , Bowman R , Donnelly R , eds (2001). Avian Ecology and Conservation in an Urbanized World. Springer, New York.

[inz212908-bib-0064] McGraw KJ (2006a). Mechanics of carotenoid‐based coloration. In: Hill GE , McGraw KJ , eds. Bird Coloration, vol. 1 *Mechanisms and Measurements*. Harvard University Press, Cambridge, MA, pp. 177–242.

[inz212908-bib-0065] McGraw KJ (2006b). Mechanisms of melanin‐based coloration. In: Hill GE, McGraw KJ, eds. Bird Coloration: Mechanisms and Measurements. Harvard University Press, Cambridge, MA, pp. 243–294.

[inz212908-bib-0066] McKinney ML (2008). Effects of urbanization on species richness: A review of plants and animals. Urban Ecosystems 11, 161–76.

[inz212908-bib-0067] McLean CA , Stuart‐Fox D , Moussalli A (2015). Environment, but not genetic divergence, influences geographic variation in colour morph frequencies in a lizard. BMC Evolutionary Biology 15, 156. 26253642 10.1186/s12862-015-0442-x PMC4528382

[inz212908-bib-0068] Megía‐Palma R , Barrientos R , Gallardo M , Martínez J , Merino S (2021). Brighter is darker: The Hamilton–Zuk hypothesis revisited in lizards. Biological Journal of the Linnean Society 134, 461–473.

[inz212908-bib-0069] Megía‐Palma R , Martínez J , Merino (2016). Structural‐ and carotenoid‐based throat colour patches in males of *Lacerta schreiberi* reflect different parasitic diseases. Behavioral Ecology and Sociobiology 70, 2017–2025.

[inz212908-bib-0070] Megía‐Palma R , Paranjpe D , Reguera S *et al*. (2018). Multiple color patches and parasites in *Sceloporus occidentalis*: differential relationships by sex and infection. Current Zoology 64, 703–711. 30538729 10.1093/cz/zoy007 PMC6280098

[inz212908-bib-0071] Mendoza‐Roldan JA , Mendoza‐Roldan MA , Otranto D (2021). Reptile vector‐borne diseases of zoonotic concern. International Journal for Parasitology: Parasites and Wildlife 15, 132–142. 34026483 10.1016/j.ijppaw.2021.04.007 PMC8121771

[inz212908-bib-0072] Merino S , Potti J (1995). High prevalence of hematozoa in nestlings of a passerine species, the pied flycatcher, *Ficedula hypoleuca* . Auk 112, 1041–1043.

[inz212908-bib-0073] Meylan S , Richard M , Bauer S , Haussy C , Miles D (2013). Costs of mounting an immune response during pregnancy in a lizard. Physiological and Biochemical Zoology 86, 127–136. 23303327 10.1086/668637

[inz212908-bib-0074] Miranda AC , Schielzeth H , Sonntag T , Partecke J (2013). Urbanization and its effects on personality traits: A result of microevolution or phenotypic plasticity? Global Change Biology 19, 2634–2644. 23681984 10.1111/gcb.12258

[inz212908-bib-0075] Nemeth E , Brumm H (2010). Birds and anthropogenic noise: Are urban songs adaptive? The American Naturalist 176, 465–475. 10.1086/656275 20712517

[inz212908-bib-0076] Nordberg EJ , Schwarzkopf L (2019). Heat seekers: A tropical nocturnal lizard uses behavioral thermoregulation to exploit rare microclimates at night. Journal of Thermal Biology 82, 107–114. 31128638 10.1016/j.jtherbio.2019.03.018

[inz212908-bib-0077] Pacyna AD , Ruman M , Mazerski J , Polkowska Ż (2018). Biological responses to environmental contamination. How can metal pollution impact signal honesty in avian species? Ecology and Evolution 8, 7733–7739. 30151185 10.1002/ece3.4192 PMC6106159

[inz212908-bib-0078] Pellitteri‐Rosa D , Bellati A , Cocca W , Gazzola A , Martín J , Fasola M (2017). Urbanization affects refuge use and habituation to predators in a polymorphic lizard. Animal Behaviour 123, 359–367.

[inz212908-bib-0079] Pérez i de Lanuza G , Font E , Carazo P (2013). Color‐assortative mating in a color‐polymorphic lacertid lizard. Behavioral Ecology 24, 273–279.

[inz212908-bib-0080] Plasman M , Reynoso VH , Nicolás L , Torres R (2015). Multiple colour traits signal performance and immune response in the Dickerson's collared lizard *Crotaphytus dickersonae* . Behavioral Ecology and Sociobiology 69, 765–775.

[inz212908-bib-0081] Prosser C , Hudson S , Thompson MB (2006). Effects of urbanization on behavior, performance, and morphology of the garden skink, *Lampropholis guichenoti* . Journal of Herpetology 40, 151–159.

[inz212908-bib-0082] Putman BJ , Gasca M , Blumstein DT , Pauly GB (2019). Downsizing for downtown: Limb lengths, toe lengths, and scale counts decrease with urbanization in western fence lizards (*Sceloporus occidentalis*). Urban Ecosystems 22, 1071–1081. 32774080 10.1007/s11252-019-00889-z PMC7409970

[inz212908-bib-0083] Putman BJ , Tippie ZA (2020). Big city living: A global meta‐analysis reveals positive impact of urbanization on body size in lizards. Frontiers in Ecology and Evolution 8, 580745.

[inz212908-bib-0084] Quinn GP , Keough MJ (2002). Experimental Design and Data Analysis for Biologists. Cambridge University Press, Cambridge.

[inz212908-bib-0085] R Develpment Core Team (2017). A Language and Environment for Statistical Computing. R Foundation for Statistical Computing, Vienna, Austria. Available from URL: http://www.R‐project.org.

[inz212908-bib-0086] Radovanović TB , Petrović TG , Gavrilović BR *et al.* (2023). What coloration brings: Implications of background adaptation to oxidative stress in anurans. Frontiers in Zoology 20, 6. 36717935 10.1186/s12983-023-00486-z PMC9887830

[inz212908-bib-0087] Riley SPD , Serieys LEK , Moriarty JG (2014). Infectious disease and contaminants in urban wildlife: Unseen and often overlooked threats. In: McCleery RA , Moorman CE , Peterson MN , eds. Urban Wildlife Conservation: Theory and Practice. Springer, Boston, MA, pp. 175–215.

[inz212908-bib-0088] Rivera‐Rea J , González‐Morales JC , Bastiaans E , Fajardo V (2018). *Sceloporus torquatus* (Torquate Lizard). Selected body temperature. Herpetological Review 49, 490.

[inz212908-bib-0089] Rivera‐Rea J , González‐Morales JC , Fajardo V , Megía‐Palma R , Bastiaans E , Manjarrez J (2022). Phenological variation in parasite load and inflammatory response in a lizard with an asynchronous reproductive cycle. The Science of Nature 109, 34 10.1007/s00114-022-01793-x 35751709

[inz212908-bib-0090] Rivera‐Rea J , González‐Morales JC , Megia‐Palma R , Bastiaans E , Quintana E , Manjarrez J (2024). Seasonal changes in color patches and parasite load of male Torquate lizard (*Sceloporus torquatus*). Behavioral Ecology and Sociobiology 78, 27.

[inz212908-bib-0091] Rogier É , Landau I (1975). Description de *Schellackia golvani* n. sp. (Lankesterellidae), parasite de Lézards de Guadeloupe. Bulletin de Muséum National d´Histoire Naturale 284, 91–97.

[inz212908-bib-0092] Roulin A (2016). Condition‐dependence, pleiotropy and the handicap principle of sexual selection in melanin‐based colouration. Biological Reviews 91, 328–348. 25631160 10.1111/brv.12171

[inz212908-bib-0093] Salathé M , Kouyos RD , Regoes RR , Bonohoeffer S (2008). Rapid parasite adaptation drive selection for high recombination rates. Evolution 62, 295–300 18039325 10.1111/j.1558-5646.2007.00265.x

[inz212908-bib-0094] Salvador A , Veiga JP , Civantos E (1999). Do skin pockets of lizards reduce the deleterious effects of ectoparasites? An experimental study with *Psammodromus algirus* . Herpetologica 1999, 1–7.

[inz212908-bib-0095] Schall JJ (1986). Prevalence and virulence of a haemogregarine parasite of the Aruban whiptail lizard, *Cnemidophorus arubensis* . Journal of Herpetology 20, 318–324.

[inz212908-bib-0096] Schall JJ (1992). Parasite‐mediated competition in *Anolis* lizards. Oecologia 92, 58–64. 28311812 10.1007/BF00317262

[inz212908-bib-0097] Schulte‐Hostedde AI , Zinner B , Millar JS , Hickling GJ (2005). Restitution of mass–size residuals: validating body condition indices. Ecology 86, 155–163.

[inz212908-bib-0098] Shuster SM (2009). Sexual selection and mating systems. PNAS 106, 10009–10016. 19528645 10.1073/pnas.0901132106 PMC2702802

[inz212908-bib-0099] Sears MW (2005). Resting metabolic expenditure as a potential source of variation in growth rates of the sagebrush lizard. Comparative Biochemistry and Physiology A 140, 171–177. 10.1016/j.cbpb.2004.12.003 15748856

[inz212908-bib-0100] Slabbekoorn H , den Boer‐Visser A (2006). Cities change the songs of birds. Current Biology 16, 2326–2331. 17141614 10.1016/j.cub.2006.10.008

[inz212908-bib-0101] Slabbekoorn H , Peet M (2003). Birds sing at a higher pitch in urban noise. Nature 424, 267–267. 10.1038/424267a 12867967

[inz212908-bib-0102] Smit JA , Cronin AD , van der Wiel I , Oteman B , Ellers J , Halfwerk W (2022). Interactive and independent effects of light and noise pollution on sexual signaling in frogs. Frontiers in Ecology and Evolution 10, 934661.

[inz212908-bib-0103] Smits JE , Bortolotti GR , Tella JL (1999). Simplifying the phytohaemagglutinin skin‐testing technique in studies of avian immunocompetence. Functional Ecology 13, 567–572.

[inz212908-bib-0104] Smyth AK , Smee E , Godfrey SS , Crowther M , Phalen D (2014). The use of body condition and haematology to detect widespread threatening processes in sleepy lizards (*Tiliqua rugosa*) in two agricultural environments. Royal Society Open Science 1, 140257. 26064571 10.1098/rsos.140257 PMC4448776

[inz212908-bib-0105] Sol D , González‐Lagos C , Lapiedra O , Díaz M (2017). Why are exotic birds so successful in urbanized environments? In: Murgui E , Hedblom M , eds. Ecology and Conservation of Birds in Urban Environments. Springer, Cham, pp. 75–89.

[inz212908-bib-0106] Sol D , Griffin AS , Bartomeus I , Boyce H (2011). Exploring or avoiding novel food resources? The novelty conflict in an invasive bird. PLoS ONE 6, e19535. 21611168 10.1371/journal.pone.0019535 PMC3097186

[inz212908-bib-0107] Sol D , Lapiedra O , González‐Lagos C (2013). Behavioural adjustments for a life in the city. Animal Behavior 85, 1101–1112.

[inz212908-bib-0108] Steiger S , Schmitt T , Schaefer HM (2011). The origin and dynamic evolution of chemical information transfer. Proceedings of the Royal Society of London B: Biological Sciences 278, 970–979. 10.1098/rspb.2010.2285 PMC3049038 21177681

[inz212908-bib-0109] Suárez‐Rodríguez M , López‐Rull I , Macías‐García C (2013). Incorporation of cigarette butts into nests reduces nest ectoparasite load in urban birds: New ingredients for an old recipe? Biology Letters 9, 20120931. 23221874 10.1098/rsbl.2012.0931 PMC3565511

[inz212908-bib-0110] Suárez‐Rodríguez M , Montero‐Montoya RD , Macías Garcia C (2017). Anthropogenic nest materials may increase breeding costs for urban birds. Frontiers in Ecology and Evolution 5, 2017.

[inz212908-bib-0111] Svensson E , Sinervo B , Comendant T (2001). Density‐dependent competition and selection on immune function in genetic lizard morphs. PNAS 98, 12561–12565. 11592973 10.1073/pnas.211071298 PMC60093

[inz212908-bib-0112] Thawley CJ , Moniz HA , Merritt AJ , Battles AC , Michaelides SN , Kolbe JJ (2019). Urbanization affects body size and parasitism but not thermal preferences in *Anolis* lizards. Journal of Urban Ecology 5, juy031.

[inz212908-bib-0113] Tucker DB , McBrayer LD , Harrison JS (2014). Population structure of Florida scrub lizard (*Sceloporus woodi*) in an anthropogenically fragment forest. Herpetologica 70, 266–278.

[inz212908-bib-0114] United Nations (2019). World Urbanization Prospects: The 2018 Revision. United Nations, New York.

[inz212908-bib-0115] Vanhooydonck B , Van Damme R (1999). Evolutionary relationship between body shape and habitat use in lacertid lizards. Evolutionary Ecology Research 1, 785–805.

