# Supplementary material for: Bluer in the city: urban male lizards exhibit more intense sexual coloration and lower parasite loads than non‐urban males
Source: Integr Zool. 2024 Sep 30;20(4):894–908. doi: 10.1111/1749-4877.12908 (PMC12235353; doi:10.1111/1749-4877.12908)
Supplement: Supplementary file 1 — Table S1 Spearman correlation results between female ventral spectral variables and parasite load [file INZ2-20-894-s001.docx]

Supplementary Table 1. Spearman correlation results between female ventral spectral variables and parasite load

| Urban females | | | |
| --- | --- | --- | --- |
|  | Mite in pockets | Total mite counts | hemoparasite load |
| Ventral luminance | 0.32 | -0.25 | -0.29 |
| Ventral UV | -0.42 | -0.12 | -0.17 |
| Ventral blue | 0.30 | -0.20 | -0.32 |
| Ventral hue | 0.22 | 0.15 | 0.37 |
|  |  |  |  |
| Non-urban females | | | |
|  | Mite in pockets | Total mite counts | hemoparasite load |
| Ventral luminance | 0.24 | 0.20 | 0.15 |
| Ventral UV | -0.09 | 0.14 | 0.50 |
| Ventral blue | 0.52 | 0.07 | -0.04 |
| Ventral hue | -0.02 | 0.27 | 0.38 |

Note: We did not find any significant relationship.
